# High HDL-C levels reduce the risk of obstructive coronary artery disease in asymptomatic diabetics who achieved optimal glycemic control

**DOI:** 10.1038/s41598-019-51732-6

**Published:** 2019-10-25

**Authors:** Gyung-Min Park, Yongjik Lee, Ki-Bum Won, Yu Jin Yang, Sangwoo Park, Soe Hee Ann, Yong-Giun Kim, Dong Hyun Yang, Joon-Won Kang, Tae-Hwan Lim, Hong-Kyu Kim, Jaewon Choe, Seung-Whan Lee, Young-Hak Kim, Shin-Jae Kim, Sang-Gon Lee

**Affiliations:** 1 Division of Cardiology, Ulsan University Hospital, University of Ulsan College of Medicine, Ulsan, Republic of Korea; 2 Division of Thoracic and Cardiovascular Surgery, Ulsan University Hospital, University of Ulsan College of Medicine, Ulsan, Republic of Korea; 3 Division of Cardiology, Asan Medical Center, University of Ulsan College of Medicine, Seoul, Republic of Korea; 4 Division of Radiology, Asan Medical Center, University of Ulsan College of Medicine, Seoul, Republic of Korea; 5 Division of Health Screening and Promotion Center, Asan Medical Center, University of Ulsan College of Medicine, Seoul, Republic of Korea

**Keywords:** Carotid artery disease, Diabetes complications

## Abstract

The benefit of a high level of high-density lipoprotein cholesterol (HDL-C) against coronary atherosclerosis risk after achieving optimal glycemic control (OGC) in diabetics remains uncertain. We aimed to evaluate the association between HDL-C and obstructive coronary artery disease (CAD) according to OGC status in diabetics. We analyzed 1,114 asymptomatic diabetics who underwent coronary computed tomographic angiography in a health examination. OGC was defined as hemoglobin A1C <7.0%. Obstructive CAD was defined as the presence of plaques with ≥50% stenosis. Patients with a high HDL-C level (≥40 mg/dL and ≥50 mg/dL in males and females, respectively) showed a lower prevalence of obstructive CAD than those with a low HDL-C level in the OGC group (8.9% vs. 14.4%; p = 0.046), but not in the non-OGC group (22.3% vs. 23.2%, p = 0.850). Multiple logistic regression models showed that the risk for obstructive CAD was lower in patients with a high HDL-C level than in those with a low HDL-C level in the OGC group (odds ratio: 0.584, 95% confidence interval: 0.343–0.995; p = 0.048), but not in the non-OGC group. In conclusion, it may be necessary to maintain a high HDL-C level to reduce the risk of obstructive CAD in asymptomatic diabetics after OGC is achieved.

## Introduction

Diabetes mellitus is significantly related to an increased risk of cardiovascular (CV) morbidity and mortality worldwide and is known to increase the risk of coronary artery disease (CAD) by two to three times^1,2^. Previous epidemiologic studies have reported that poor glycemic control significantly influences the risk of major CV events^3–5^. In addition, large-scale longitudinal studies with long-term follow-up have revealed the efficacy of glycemic control in reducing adverse CV outcomes^6,7^. Therefore, achieving optimal glycemic control (OGC) has been emphasized as the main therapeutic strategy in patients with established diabetes.

Although several studies have suggested the significance of high-density lipoprotein cholesterol (HDL-C) in the prevention of atherosclerosis^8–10^, the clinical benefit of an elevated HDL-C level is still controversial^11–14^. In particular, there is a paucity of data regarding whether an association exists between the HDL-C level and CAD severity after OGC is achieved in patients with established diabetes. Coronary computed tomography angiography (CCTA) has been recently shown to be an effective non-invasive imaging tool with high diagnostic performance in the detection of CAD and provides incremental prognostic utility in predicting major adverse CV outcomes^15–18^. Therefore, the present study aimed to evaluate the relationship between the HDL-C level and obstructive CAD on CCTA according to OGC status in asymptomatic patients with diabetes.

## Methods

### Study population

Initially, 9,269 consecutive South Korean patients, aged ≥20 years, underwent a self-referred CCTA evaluation as part of a general health examination at the Asan Medical Center between January 2007 and December 2011. The potential risks of CCTA were explained, and written informed consent was obtained from all patients included in this study. The study exclusion criteria were as follows: (1) refusal to take part in the study (n = 2140); (2) history of angina or myocardial infarction (n = 336); (3) abnormal electrocardiographic findings, including left bundle branch block, pathologic Q wave, or ischemic changes in the ST segments or T wave (n = 205); (4) insufficient medical records (n = 85); (5) structural heart disease (n = 49); (6) history of percutaneous coronary intervention (n = 5) or open heart surgery (n = 5); (7) history of cardiac procedures, including atrial septal defect device closure (n = 4), percutaneous mitral valvuloplasty (n = 2), permanent pacemaker implantation (n = 2), patent ductus arteriosus device closure (n = 1), and patent foramen ovale device closure (n = 1); (8) renal insufficiency (n = 1); and (9) no evidence of diabetes (n = 5,319). Finally, a total of 1,114 patients with diabetes were enrolled in this single-center, retrospective, and observational study. The study was approved by the local Institutional Review Board of the Asan Medical Center, Seoul, Korea.

Clinical information was collected from the Health Screening and Promotion Center at the Asan Medical Center. Medical histories were obtained during the general health examination via self-reported questionnaires. A family history of CAD was defined as having a first-degree relative, of any age, with CAD. Diabetes was defined as a fasting glucose level ≥126 mg/dL, hemoglobin A1C (HbA1C) level ≥6.5%, or anti-diabetic medication use. OGC was defined as an HbA1C level <7.0%. Hypertension was defined as systolic/diastolic blood pressures ≥ 140/90 mmHg, or anti-hypertensive medication use. Hyperlipidemia was defined as a total cholesterol level ≥240 mg/dL or anti-hyperlipidemic medication use. High triglyceride was defined as a triglyceride level ≥150 mg/dL. High HDL-C was defined as an HDL-C level ≥40 mg/dL and ≥50 mg/dL in males and females, respectively. High low-density lipoprotein cholesterol (LDL-C) was defined as an LDL-C level ≥100 mg/dL. Using the Framingham risk score, the 10-year risk for CAD was categorized as low (<10%), intermediate (10–20%), or high (>20%)^19^. All methods were performed in accordance with the relevant guidelines and regulations.

### Acquisition and analysis of CCTA images

CCTA was performed using a dual-source (Somatom Definition, Siemens, Erlangen, Germany) or single-source 64-slice computed tomography (CT) system (LightSpeed VCT, GE, Milwaukee, WI, USA). Patients without an absolute contraindication to beta-blockers and with a heart rate >65 bpm received 2.5 mg bisoprolol (Concor, Merck, Darmstadt, Germany) 1 hour before the examination. The prospective electrocardiography (ECG)-triggering mode or retrospective ECG-gating mode was used during CT scanning, with ECG-based current modulation. Before contrast injection, two puffs (2.5 mg) of isosorbide dinitrate (Isoket spray, Schwarz Pharma, Monheim, Germany) were administered. During CCTA acquisition, iodinated contrast (60–80 mL; Imeron® 400, Bracco, Milan, Italy) was injected at 4 mL/s, followed by a flush of saline (40 mL). A region of interest was placed in the ascending aorta, and the acquisition of images was initiated once the threshold of 100 HU had been reached using bolus tracking. A standard CT scan protocol was used (tube voltage, 100 or 120 kVp; tube current, 240–400 mAs per rotation for dual-source CT and 400–800 mA for 64-slice CT), and the product of the tube voltage and tube current time was adjusted in consideration of the patient’s body size. The size-specific dose was calculated using the patient’s body diameter^20^. The effective dose of the CT protocol was 9.0 ± 4.5 mSv.

All images were analyzed on workstations (Advantage Workstation, GE; or Volume Wizard, Siemens) by experienced cardiovascular radiologists (D.H.Y., J.-W.K., and T.-H.L.) in accordance with the guidelines of the Society of Cardiovascular Computed Tomography^21^. The coronary artery calcium score (CACS) was calculated as previously described^22^. Plaques, defined as structures ≥1 mm^2^ in size, within or adjacent to the vessel lumen, were clearly distinguishable from the lumen and surrounding pericardial tissue. Plaques with calcium (>130 HU) involving ≥\50% of the plaque area were classified as calcified, those with <50% calcium were classified as mixed, and those without calcium were classified as non-calcified^23^. The contrast-enhanced luminal portion was semi-automatically traced at the site of maximal stenosis and compared with the mean value of the proximal and distal reference sites^24^. Luminal stenosis ≥50% was defined as obstructive. Obstructive CAD was defined as the presence of an obstructive plaque.

### Statistical analysis

Continuous variables are expressed as means ± standard deviation (SD), and categorical variables are expressed as absolute values and proportions. Group differences in continuous variables were evaluated using the independent *t*-test or Mann-Whitney U test, in accordance with the normality status of the data distribution. Categorical variables were compared using the chi-square test or Fisher’s exact test, as appropriate. Univariate and multivariate logistic regression analyses were performed to evaluate relationships between clinical variables and obstructive CAD in the total population. Subsequently, multiple logistic regression models were used to identify the impact of a high HDL-C level on obstructive CAD according to OGC status. Prior to performing the logistic regression analyses, the absence of multicollinearity among the independent variables was confirmed via correlation analyses. The forced entry method was used to enter independent variables into the multiple regression analysis. All statistical analyses were performed using the Statistical Package for the Social Sciences version 19 (SPSS, Chicago, Illinois). A p-value <0.05 was considered significant in all analyses.

### Ethics approval and consent to participate

The protocol of the present study was approved by the institutional review board of the Asan Medical Center, and written informed consent was obtained from each participant.

## Results

### Baseline characteristics

The baseline characteristics of the total study population are presented in Table 1. The mean age was 55.8 ± 7.5 years, and 921 patients (82.7%) were male. The mean fasting glucose and HbA1C levels were 134.0 ± 32.5 mg/dL and 6.8% ± 1.2% (51.3 ± 13.1 mmol/mol), respectively. Among the patients, 67.0% had achieved OGC. Hypertension, hyperlipidemia, and obesity were observed in 52.0%, 44.3%, and 53.7% of the patients, respectively. Among the patients, 30.8% were current smokers. The mean total cholesterol, triglyceride, HDL-C, and LDL-C levels were 186.6 ± 38.6 mg/dL, 159.3 ± 115.7 mg/dL, 50.3 ± 12.3 mg/dL, and 112.8 ± 32.9 mg/dL, respectively. High levels of triglyceride, HDL-C, and LDL-C were observed in 40.0%, 77.4%, and 64.0% of the patients, respectively. According to the CCTA findings, the mean CACS was 78.0 ± 194.6. Any plaque and obstructive plaque were observed in 57.8% and 14.2% of the patients, respectively.

**Table 1:** Baseline characteristics of the total study population.

Characteristics	n = 1,114
**Clinical data**	
Age, year	55.8 ± 7.5
Male, n (%)	921 (82.7)
Systolic blood pressure, mmHg	123.3 ± 13.7
Diastolic blood pressure, mmHg	78.2 ± 10.1
BMI, kg/m^2^	25.4 ± 3.0
Obesity, n (%)	598 (53.7)
Hypertension, n (%)	579 (52.0)
Hyperlipidemia, n (%)	494 (44.3)
Current smoking, n (%)	338 (30.8)
Family history of CAD, n (%)	137 (12.3)
Framingham risk score	12.0 ± 6.0
Framingham risk stratification, n (%)	
Low	483 (43.4)
Intermediate	514 (46.1)
High	117 (10.5)
Total cholesterol, mg/dL	186.6 ± 38.6
Triglyceride, mg/dL	159.3 ± 115.7
HDL-C, mg/dL	50.3 ± 12.3
LDL-C, mg/dL	112.8 ± 32.9
High triglyceride, n (%)	446 (40.0)
High HDL-C, n (%)	862 (77.4)
High LDL-C, n (%)	713 (64.0)
Fasting glucose, mg/dL	134.0 ± 32.5
HbA1C, %	6.8 ± 1.2
Creatinine, mg/dL	0.9 ± 0.2
Anti-hypertensive treatment, n (%)	467 (41.9)
Anti-hyperlipidemic treatment, n (%)	291 (26.1)
Anti-diabetic treatment, n (%)	576 (51.7)
OGC, n (%)	746 (67.0)
**CCTA data**	
CACS	78.0 ± 194.6
Any plaque, n (%)	644 (57.8)
Calcified plaque, n (%)	468 (42.0)
Non-calcified plaque, n (%)	277 (24.9)
Mixed plaque, n (%)	178 (16.0)
Obstructive plaque, n (%)	158 (14.2)

Values are presented as means ± standard deviation or as numbers (%).

*BMI* body mass index, *CACS* coronary artery calcium score, *CAD* coronary artery disease, *CCTA* coronary computed tomographic angiography, *HDL-C* high-density lipoprotein cholesterol, *LDL-C* low-density lipoprotein cholesterol, *OGC* optimal glycemic control.

### Prevalence of obstructive CAD according to OGC status and the level of each lipid

**Figure 1:**
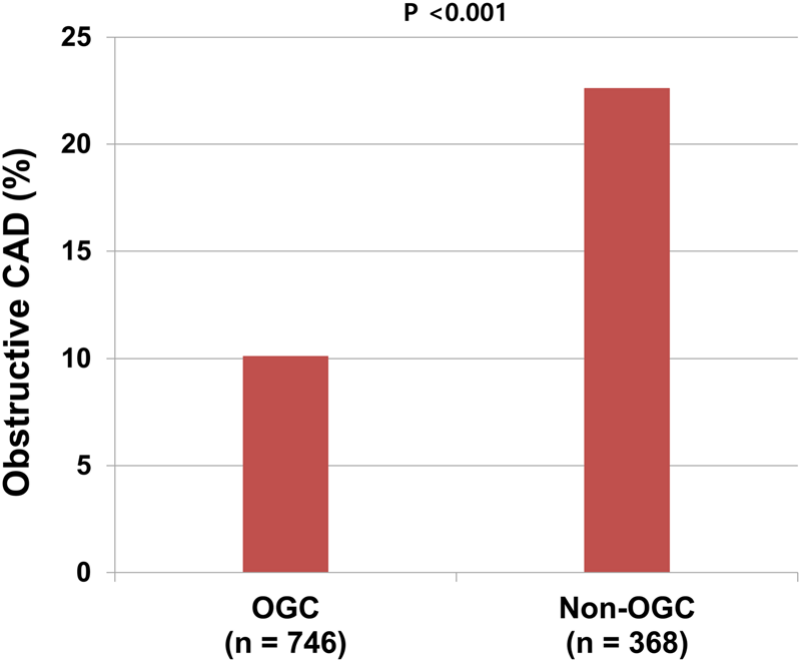
Prevalence of obstructive CAD according to OGC status. *CAD* coronary artery disease, *OGC* optimal glycemic control.

**Figure 2:**
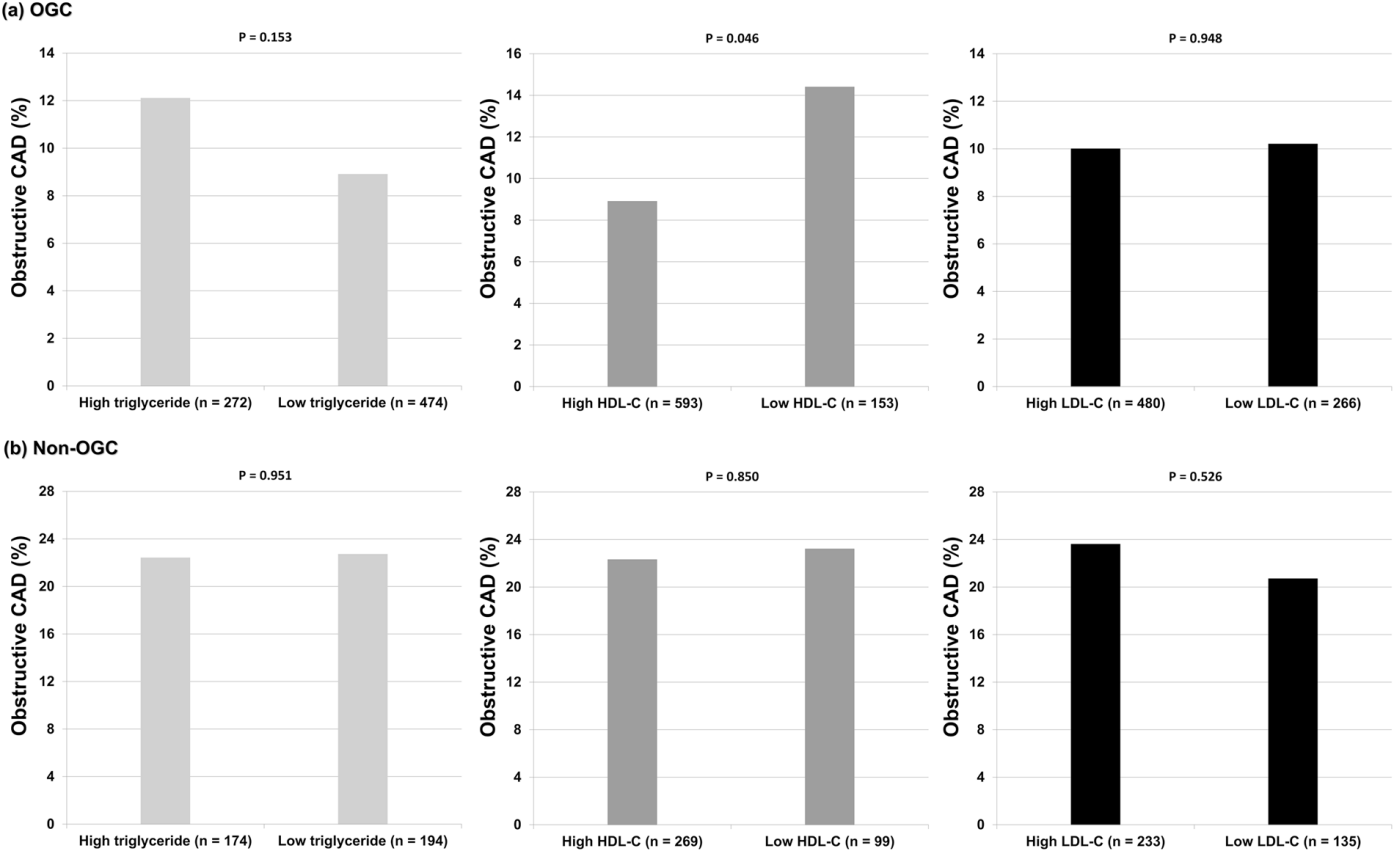
Prevalence of obstructive CAD in the OGC and non-OGC groups, according to the level of each lipid. *CAD* coronary artery disease, *OGC* optimal glycemic control.

A significantly lower prevalence of obstructive CAD was observed in the OGC group than in the non-OGC group (10.1% vs. 22.6%; p < 0.001) (Fig. 1). In the OGC group, patients with a high HDL-C level had a lower prevalence of obstructive CAD than did those with a low HDL-C level (8.9% vs. 14.4%; p = 0.046); however, the prevalence of obstructive CAD did not significantly differ between patients with and without a high level of triglyceride (12.1% vs. 8.9%; p = 0.153) or LDL-C (10.0% vs. 10.1%; p = 0.948). In the non-OGC group, the prevalence of obstructive CAD did not significantly differ between patients with and without a high level of triglyceride (22.4% vs. 22.7%; p = 0.951), HDL-C (22.3% vs. 23.2%; p = 0.850), or LDL-C (23.6% vs. 20.7%; p = 0.526) (Fig. 2).

### Associations between clinical variables and obstructive CAD in the total study population

In the univariate logistic regression analysis, age (odds ratio [OR]: 1.062; 95% confidence interval [CI]: 1.039–1.087; p < 0.001), hypertension (OR: 1.516; 95% CI: 1.075–2.138; p = 0.018), and HbA1C level (OR: 1.383; 95% CI: 1.227–1.559; p < 0.001) were associated with an increased risk of obstructive CAD. In the multivariate logistic regression analysis, age (OR: 1.082; 95% CI: 1.055–1.111; p < 0.001), a family history of CAD (OR: 1.817; 95% CI: 1.101–2.997; p = 0.019), and HbA1C level (OR: 1.440; 95% CI: 1.264–1.640; p < 0.001) were independently associated with an increased risk of obstructive CAD (Table 2).

**Table 2:** Associations between clinical variables and obstructive CAD in the total study population.

Variables	Univariate		Multivariate	
	OR (95% CI)	p	OR (95% CI)	p
Age, per 1 year	1.062 (1.039–1.087)	<0.001	1.082 (1.055–1.111)	<0.001
Male sex	1.134 (0.717–1.794)	0.590	1.584 (0.949–2.643)	0.078
BMI, per 1 kg/m^2^	1.007 (0.951–1.065)	0.814	1.011 (0.949–1.078)	0.728
Hypertension	1.516 (1.075–2.138)	0.018	1.383 (0.948–2.017)	0.092
Triglyceride, per 1 mg/dL	1.001 (0.999–1.001)	0.241	1.001 (0.999–1.002)	0.273
HDL-C, per 1 mg/dL	0.986 (0.971–1.000)	0.053	0.989 (0.973–1.005)	0.181
LDL-C, per 1 mg/dL	1.001 (0.996–1.006)	0.796	1.002 (0.996–1.007)	0.518
Current smoking	1.002 (0.695–1.445)	0.991	1.129 (0.748–1.704)	0.563
Family history of CAD	1.416 (0.885–2.258)	0.147	1.817 (1.101–2.997)	0.019
HbA1C, %	1.383 (1.227–1.559)	<0.001	1.440 (1.264–1.640)	<0.001

*BMI* body mass index, *CAD* coronary artery disease, *CI* confidence interval, *HbA1C* hemoglobin A1C, *OR* odds ratio, *HDL-C* high-density lipoprotein cholesterol, *LDL-C* low-density lipoprotein cholesterol.

### Impact of a high HDL-C level on obstructive CAD according to OGC status

In the multiple logistic regression models, a high HDL-C level was independently associated with a decreased risk of obstructive CAD after consecutive adjustment for age, sex, obesity, hypertension, current smoking, triglyceride level, LDL-C level, and a family history of CAD in the OGC group. However, a high HDL-C level was not significantly associated with obstructive CAD in the non-OGC group (Table 3).

**Table 3:** Impact of a high level of high-density lipoprotein cholesterol on obstructive CAD according to OGC status.

Variables	OR (95% CI)	P
OGC group		
Model 1	0.584 (0.343–0.995)	0.048
Model 2	0.485 (0.277–0.851)	0.012
Model 3	0.514 (0.290–0.909)	0.022
Model 4	0.525 (0.292–0.943)	0.031
Non-OGC group		
Model 1	0.949 (0.549–1.640)	0.850
Model 2	0.931 (0.526–1.647)	0.806
Model 3	0.903 (0.506–1.612)	0.731
Model 4	0.919 (0.507–1.663)	0.779

*BMI* body mass index, *CAD* coronary artery disease, *CI* confidence interval, *HbA1C* hemoglobin A1C, *OGC* optimal glycemic control, *OR* odds ratio.

Model 1: Unadjusted.

Model 2: Adjusted for age and sex.

Model 3: Adjusted for age, sex, obesity, hypertension, and current smoking.

Model 4: Adjusted for age, sex, obesity, hypertension, current smoking, triglyceride level, low-density lipoprotein cholesterol level, and a family history of CAD.

## Discussion

Consistent with previous studies, OGC status had a close association with coronary atherosclerosis in patients with established diabetes in the present study. However, to the best of our knowledge, the present study is the first to reveal that a high HDL-C level is independently associated with a decreased risk of obstructive CAD in patients with diabetes who have achieved OGC.

The Aerobics Center Longitudinal Study (ACLS) previously reported that diabetes is associated with a 3-fold greater risk for CV mortality, and that metabolic syndrome, with a clustering of CV risk factors, did not affect this risk^25^. Won *et al*. also reported that metabolic syndrome did not have a significant association with subclinical atherosclerosis, as reflected in the brachial-ankle pulse wave velocity, carotid intima-media thickness, and carotid plaque, in established diabetics from a large cross-sectional cohort study^26^. However, these studies are limited in that the glycemic control status of the patients with diabetes was not considered. Recent long-term follow-up studies have suggested that hyperglycemia directly influences the prognosis of patients with established diabetes^6,7^. In addition, several longitudinal studies have reported that poor glycemic control is related to the progression of coronary calcification in patients with established diabetes^27,28^. Despite strong evidence for the significance of glycemic control, there is a paucity of data regarding associations between metabolic abnormalities and the severity of coronary atherosclerosis after OGC is achieved in the asymptomatic diabetic population. In the present study, the prevalence of obstructive CAD in the OGC group was less than half of that in the non-OGC group. Furthermore, we found a beneficial effect of a high HDL-C level on obstructive CAD in only the OGC group, after adjusting for traditional risk factors. Considering that myocardial ischemia is often asymptomatic and frequently manifests clinically in advanced stages in patients with diabetes^29,30^, the maintenance of a high HDL-C level in the asymptomatic diabetic population might be necessary to reduce the risk of obstructive CAD after achieving OGC.

Current American Diabetes Association guidelines mainly recommend lowering the LDL-C level with statins, the dose of which is determined by atherosclerotic CV risk factors, rather than the LDL-C level alone, as the primary target of hyperlipidemia treatment in the diabetic population^31^. In the present study, the LDL-C level did not differ according to OGC status (OGC group: 112.6 ± 32.4 mg/dL vs. non-OGC: 113.1 ± 33.8; p = 0.809). In addition, there were no significant differences in the prevalence of obstructive CAD between patients with and without high triglyceride and LDL-C levels. Although an independent relationship between the HDL-C level and obstructive coronary plaques in patients with diabetes who had achieved OGC was revealed in the present study, the effect of specific lipid-lowering agents and doses on coronary atherosclerosis was not evaluated. Further large-scale prospective investigations are necessary to investigate this aspect.

The present study has several limitations. First, all patients voluntarily participated in a general health examination. Therefore, a selection bias might exist. Second, we evaluated obstructive CAD focusing only on each cholesterol level because anti-hyperlipidemic agents were not controlled in this observational study. Third, high-risk plaque features, including positive remodeling of the coronary artery, low-attenuation plaque, and the napkin-ring sign^32,33^, were not analyzed in the present study. Finally, although our study only enrolled volunteers, CCTA is not currently recommended in asymptomatic individuals, despite advancements in CCTA techniques^34^. Despite these limitations, the present study is unique in that we identified an independent relationship between a high HDL-C level and obstructive CAD after achieving OGC in the asymptomatic diabetic population.

## Conclusions

Beyond the importance of achieving OGC for the prevention of coronary atherosclerosis, the present study revealed the importance of maintaining a high HDL-C level to reduce the risk of obstructive CAD in asymptomatic diabetics once OGC is achieved. Further randomized and prospective studies with large sample sizes are necessary to confirm these results.

## Data Availability

The datasets used and analyzed during the current study are available from the corresponding author on reasonable request.
